# HIV and Cancer: Insights into Viral-Mediated Oncogenesis and Immunosuppression

**DOI:** 10.3390/pathogens15040416

**Published:** 2026-04-12

**Authors:** Angioletta Lasagna, Giacomo Pozza, Maddalena Matone, Cinzia Fasola, Lorenzo Ruggieri, Nicla La Verde, Paolo Pedrazzoli, Davide Dalu

**Affiliations:** 1 Department of Oncology, Comprehensive Cancer Center, Fondazione IRCCS Policlinico San Matteo, 27100 Pavia, Italy; 2 Department of Infectious Diseases, Luigi Sacco Hospital, ASST Fatebenefratelli Sacco, 20100 Milan, Italymatone.maddalena@asst-fbf-sacco.it (M.M.);; 3 Infectious Disease Oncology Unit, Department of Oncology, Luigi Sacco Hospital, ASST Fatebenefratelli Sacco, 20100 Milan, Italy; ruggieri.lorenzo@asst-fbf-sacco.it (L.R.); davide.dalu@asst-fbf-sacco.it (D.D.); 4 Department of Internal Medicine and Medical Therapy, University of Pavia, 27100 Pavia, Italy

**Keywords:** oncogenic virus, immune cell exhaustion, immunosenescence, HHV-8

## Abstract

Background: People living with HIV (PLWH) have a substantially increased risk of both AIDS-defining cancers (ADCs) and non-AIDS-defining cancers (NADCs), which remain a major cause of morbidity despite effective antiretroviral therapy (ART); this review aims to integrate current epidemiological, molecular, and clinical evidence on HIV-associated oncogenesis. Methods: A structured literature search was conducted in PubMed (2000–2026) using predefined keywords, including “HIV”, “cancer”, “oncogenesis”, and “immune dysregulation”, with inclusion of original studies, systematic reviews, and meta-analyses meeting predefined quality criteria. Results: Available evidence indicates that HIV contributes to cancer development through both direct and indirect mechanisms: viral proteins such as Tat, Nef, and Vpr disrupt apoptosis, DNA repair, and cell cycle regulation, while chronic immune activation, persistent inflammation, and immunosuppression impair tumor immune surveillance and facilitate oncogenic viral co-infections, including Epstein–Barr virus, human papillomavirus, and human herpesvirus 8. Emerging pathways, such as epigenetic alterations, microRNA dysregulation, metabolic reprogramming, and the contribution of HIV reservoirs to pro-tumorigenic microenvironments, further modulate cancer risk. Conclusions: HIV may function as a cofactor that enhances the effects of oncogenic viruses by promoting viral persistence and immune dysregulation; while biologically plausible, direct evidence linking HIV to amplification of tumorigenesis in humans remains limited.

## 1. Introduction

HIV infection significantly increases cancer risk in people living with HIV (PLWH), posing challenges that span epidemiology, molecular mechanisms, and clinical management. Even in the era of effective antiretroviral therapy (ART), which has improved life expectancy and reduced HIV-related morbidity, PLWH remain at higher risk of both AIDS-defining cancers (ADCs) and non-AIDS-defining cancers (NADCs) [1,2,3]. Classic ADCs—Kaposi’s sarcoma (KS), non-Hodgkin lymphoma (NHL), and invasive cervical carcinoma—are closely linked to immunodeficiency and opportunistic viral co-infections such as HHV-8, EBV, and HPV [4,5,6,7]. NADCs, including cancers of the lung, liver, anus, head and neck, and Hodgkin lymphoma, have become increasingly prevalent with longer survival, influenced by chronic inflammation, immune dysregulation, lifestyle factors, and co-infections [3,8,9,10,11].

HIV contributes to oncogenesis both directly, via viral proteins Tat, Nef, and Vpr that disrupt apoptosis, proliferation, and DNA repair, and indirectly, through immune dysfunction and chronic inflammation, which facilitate persistent infection with oncogenic viruses [12]. Effective ART reduces some ADCs by restoring immune function, yet the risk of NADCs persists, underscoring the importance of screening, vaccination, and lifestyle interventions [13].

This review integrates epidemiological, mechanistic, and clinical insights, highlighting both established and emerging pathways of HIV-associated oncogenesis, including direct viral effects on epithelial and immune cells and the role of chronic immune activation in potentiating oncogenic co-infections.

## 2. Materials and Methods

This review was conducted as a structured narrative review with systematic search elements. A structured literature search was performed in PubMed using predefined keywords and their combinations (including “HIV”, “cancer”, “oncogenesis”, “immune dysregulation”, “AIDS-defining cancers”, and “non-AIDS-defining cancers”), applying Boolean operators. Two authors to minimize selection bias independently carried out the screening process, and any discrepancies were resolved through discussion. Searches were limited to publications in English from 2000 to the inception of 2026. We selected the year 2000 as the starting point for our literature search because it corresponds to the early phase of the widespread introduction of combination ART. By focusing on studies published from 2000 onward, we aimed to capture data that are more representative of the modern ART era, in which immune reconstitution, chronic inflammation, and NADCs play a central role.

Inclusion criteria were original research, systematic reviews, and meta-analyses reporting epidemiological, mechanistic, or clinical data on HIV-related malignancies. Exclusion criteria included non-human studies not relevant to HIV pathogenesis, conference abstracts without full data, and studies with insufficient methodological detail. All retrieved records were screened for duplicates and independently assessed by two authors. Quality assessment was performed using adapted criteria derived from established methodological frameworks for epidemiological and mechanistic studies, including sample size, study design, and clarity of reported outcomes. These criteria were tailored to the scope of this review and applied using predefined internal thresholds. Discrepancies were resolved through discussion. Only studies meeting predefined quality thresholds were included in the final synthesis.

## 3. Direct Role of HIV in Carcinogenesis

Although HIV is not a classical oncogenic virus, the IARC classifies it as Group 1 for HIV-1 (carcinogenic to humans) and Group 2B for HIV-2 (possibly carcinogenic to humans) [14]. Increasing experimental and clinical evidence supports a direct contribution of HIV to carcinogenesis through the biological activities of its viral proteins. While early preclinical research identified HIV-1 replication within human uterine epithelial cells, subsequent evidence has largely failed to demonstrate similar productive infection in other epithelial tissues. Instead, the consensus across a broad range of data suggests that while epithelial cells of various origins can sequester HIV-1, they do not typically support active viral replication or genomic integration. Recent findings, however, have highlighted a specific pathway for viral entry: HIV-infected T cells can fuse with epithelial cells that express syncytin—an envelope glycoprotein derived from the human endogenous retrovirus family W1. This fusion-mediated transfer bypasses standard entry barriers, effectively establishing the epithelial layer as a persistent HIV-1 reservoir [15]. Unlike oncogenic viruses that induce malignant transformation via insertional mutagenesis or viral oncogenes, HIV promotes tumor development primarily by modulating host cellular pathways involved in transcriptional regulation, apoptosis, DNA repair, and immune signaling [12,16,17]. Among HIV-encoded proteins, Tat (trans-activator of transcription), Nef (negative regulatory factor), and Vpr (viral protein R) have been most extensively implicated in these processes and are increasingly recognized as key mediators of HIV-associated oncogenic effects [18]. The HIV transactivator protein Tat plays a central role in viral replication but also exerts pleiotropic effects on host cells. Tat is actively secreted by infected cells and can be taken up by neighboring uninfected cells, thereby extending its biological impact beyond HIV-infected targets. At the molecular level, Tat functions as a potent transcriptional regulator by interacting with host transcription factors such as NF-κB, Sp1, and AP-1, leading to dysregulated expression of genes involved in cell proliferation, angiogenesis, and survival [19]. Tat has been shown to upregulate pro-angiogenic factors including vascular endothelial growth factor (VEGF) [20] and basic fibroblast growth factor (bFGF) [21], contributing to the angiogenic phenotype characteristic of certain HIV-associated malignancies, most notably Kaposi’s sarcoma [22,23]. In addition, Tat interferes with apoptotic pathways by modulating p53 function and altering the balance between pro- and anti-apoptotic proteins, thereby promoting cell survival under genotoxic stress [24]. Evidence also suggests that Tat impairs DNA repair mechanisms, increasing genomic instability and susceptibility to malignant transformation [25]. Nef, another multifunctional HIV accessory protein, contributes to oncogenesis through its ability to alter intracellular signaling pathways and immune cell function. Nef is expressed early during infection and is essential for viral pathogenicity. It interacts with multiple host proteins involved in signal transduction, including Src family kinases and components of the PI3K/Akt pathway, leading to enhanced cell activation and survival. In immune cells, Nef promotes chronic activation while simultaneously impairing effective immune responses by downregulating major histocompatibility complex class I (MHC-I) molecules, thus facilitating immune evasion [26,27]. This paradoxical combination of immune activation and immune escape creates a permissive environment for malignant progression. Furthermore, Nef has been shown to induce oxidative stress and alter cytoskeletal dynamics, processes that may contribute to genomic instability and tumor cell invasion [28]. Finally, Nef can modulate the tumor microenvironment by influencing cytokine secretion and intercellular communication, indirectly supporting tumor growth and persistence [29]. Vpr is another HIV protein implicated in direct oncogenic processes and is known for its ability to induce cell cycle arrest at the G2/M checkpoint, a state that favors viral replication but also has profound implications for genomic integrity [30,31]. Prolonged G2 arrest is associated with impaired DNA damage response and accumulation of chromosomal abnormalities, both of which are hallmarks of cancer development [32]. Vpr also induces DNA double-strand breaks and interferes with DNA repair pathways, further contributing to genomic instability [33]. HIV-1 Vpr modulates apoptotic signaling in a manner that depends on both intrinsic cellular properties and extrinsic environmental cues. In some cell types, Vpr promotes apoptosis, particularly when DNA damage responses and pro-apoptotic pathways such as p53 and Bax are intact. In other cells, especially those exposed to chronic inflammatory stimuli or with elevated survival signaling through pathways like NF-κB and Bcl-2, Vpr can enhance cell survival despite the presence of genotoxic stress. This context-dependent modulation of apoptosis allows damaged or genetically unstable cells to persist and proliferate, potentially facilitating the emergence and expansion of cells with malignant potential [34]. Beyond their direct effects on individual cells, HIV proteins exert broader influences on the immune system and the tumor microenvironment. Chronic exposure to Tat, Nef, and Vpr contributes to sustained immune activation, cytokine dysregulation, and remodeling of tissue microenvironments. HIV proteins have been shown to induce the production of pro-inflammatory cytokines such as TNF-α, IL-6, and IL-8, which are known to promote tumor initiation and progression through activation of oncogenic signaling pathways including NF-κB and STAT3. Persistent cytokine release fosters a pro-tumorigenic inflammatory milieu that supports angiogenesis, immune evasion, and tumor cell survival [35,36,37]. Moreover, HIV proteins affect the function of key immune cell populations involved in tumor surveillance. Tat and Nef impair dendritic cell maturation and antigen presentation, reducing the ability of the immune system to recognize and eliminate transformed cells [38]. Natural killer (NK) cell function is also compromised, further weakening antitumor immunity [39]. In parallel, HIV infection induces profound functional alterations in both macrophages and T cells, reshaping the local immune landscape and promoting the establishment of an immunosuppressive tumor microenvironment. In macrophages, HIV can drive polarization towards a regulatory or M2-like phenotype, characterized by secretion of immunosuppressive cytokines such as IL-10 and TGF-β, as well as impaired antigen presentation [40]. Importantly, the direct oncogenic effects of HIV proteins may act synergistically with other cancer-promoting factors present in people living with HIV, including co-infections with oncogenic viruses, environmental exposures, and aging-related immune dysfunction. By altering cellular signaling, transcriptional programs, and immune regulation, HIV proteins lower the threshold for malignant transformation and support tumor progression even in the absence of direct viral integration into tumor cells [41]. This concept helps explain why certain malignancies occur at higher rates in PLWH individuals despite effective suppression of viral replication with antiretroviral therapy. Table 1 summarizes the experimental, translational, and clinical evidence regarding the oncogenic potential of HIV proteins Tat, Nef, and Vpr.

## 4. Indirect Role of HIV in Carcinogenesis

Beyond direct effects of viral proteins, HIV promotes cancer indirectly through a combination of immune dysfunction, chronic inflammation, and opportunistic co-infections, all of which reshape the host environment to favor malignant transformation. A central mechanism is immunosuppression and impaired immune surveillance. HIV preferentially depletes CD4^+^ T cells, particularly naïve and central memory subsets, leading to a compromised adaptive immune system. Concurrently, chronic HIV infection causes functional exhaustion of CD8^+^ cytotoxic T cells and NK cells, reducing their ability to recognize and eliminate transformed or virus-infected cells [42]. Dendritic cells and macrophages also display impaired antigen presentation and altered cytokine production, further weakening immune-mediated tumor control [43]. As a result, early neoplastic cells can persist, accumulate genetic lesions, and evade immune clearance—a prerequisite for tumor initiation. Expansion of Tregs and myeloid-derived suppressor cells (MDSCs) under HIV infection enhances this immunosuppressive environment, as these populations secrete inhibitory cytokines such as IL-10 and TGF-β, which dampen effector T cell responses and create a tumor-permissive microenvironment [40].

Persistent infection with human papillomavirus (HPV), Epstein–Barr virus (EBV), and human herpesvirus 8 (HHV-8) is more common in HIV-positive individuals [44,45,46]. Impaired immune clearance allows viral oncoproteins—such as HPV E6/E7 [47], EBV LMP1/LMP2 [48], and HHV-8 vFLIP [48]—to remain active, driving proliferation, inhibiting apoptosis, and reprogramming host transcriptional networks. These viruses synergize with HIV-induced inflammation: NF-κB and STAT3 activation induced by HIV proteins enhance viral gene expression and promote a pro-tumorigenic milieu. For instance, in HPV-associated anal or cervical lesions, HIV-driven immunosuppression allows persistence of high-risk HPV strains, while inflammatory cytokines potentiate the survival of transformed epithelial cells [49]. Similarly, EBV-driven lymphomas occur more frequently in PLWH hosts due to diminished cytotoxic control and chronic immune activation [50]. The interplay between immunosuppression, inflammation, and co-infection creates a self-reinforcing loop. HIV-mediated immune dysfunction enables oncogenic viruses to establish persistent infection, which in turn sustains inflammation and tissue remodeling. Continuous NF-κB and STAT3 signaling maintains pro-survival and proliferative programs in both infected and adjacent cells, while Tregs and MDSCs limit immune-mediated tumor suppression. Collectively, these indirect effects of HIV—without direct integration into tumor genomes—lower the threshold for malignant transformation and facilitate tumor progression across multiple tissues [37]. Epidemiological evidence supports this mechanistic framework: NADCs, including lung, liver, anal, and Hodgkin lymphoma, remain elevated in PLWH population despite viral suppression with ART [51]. These cancers are rarely directly infected by HIV but are strongly influenced by chronic immune activation, dysregulated cytokine networks, and co-infection with oncogenic viruses.

## 5. Emerging Molecular Mechanisms in HIV-Associated Oncogenesis

Recent evidence highlights the contribution of emerging molecular mechanisms to HIV-associated cancer development, expanding our understanding beyond direct viral protein effects and immune dysregulation. Among these, microRNAs (miRNAs) and epigenetic modifications have gained increasing attention. HIV infection can dysregulate host miRNA networks, either through direct viral effects or chronic immune activation, altering gene expression programs that control cell proliferation, apoptosis, and DNA repair. For instance, specific miRNAs modulated by HIV or co-infecting oncogenic viruses can act as oncogenes or tumor suppressors, influencing the susceptibility of host cells to malignant transformation [52]. Similarly, epigenetic alterations, including DNA methylation and histone modifications, have been observed in HIV-infected cells and tissues, potentially facilitating oncogenic transcriptional programs and persistent viral latency, which may cooperate with other oncogenic stimuli [53]. Another layer of HIV-associated oncogenic risk involves metabolic reprogramming and changes in the tumor microenvironment. Chronic HIV infection and immune activation induce metabolic alterations in immune and epithelial cells, including shifts in glycolysis, oxidative phosphorylation, and lipid metabolism, which can create a pro-survival and pro-proliferative environment [54]. Interactions between altered metabolism, inflammatory mediators, and co-infecting oncogenic viruses further amplify the risk of malignant transformation and tumor progression. These emerging mechanisms also hold promise for identifying novel biomarkers of oncogenic risk in people living with HIV. miRNA profiles, epigenetic signatures, and metabolic markers may serve as predictive or prognostic indicators, enabling risk stratification and early intervention. Integrating these molecular insights with clinical and immunological data could facilitate personalized screening strategies and targeted preventive approaches, ultimately improving outcomes in PLWH patients at risk for both ADCs and NADCs.

## 6. Viral Synergism: HIV as an Amplifier of Oncogenic Viruses

Although HIV is not a classical transforming virus, its effects on immune regulation and tissue homeostasis may create an environment that facilitates the oncogenic potential of persistent viral infections. Rather than acting as a direct carcinogen, HIV could serve as a cofactor by promoting viral persistence, sustaining chronic inflammation, and indirectly contributing to conditions favorable for malignant transformation [7]. Chronic HIV infection disrupts immune surveillance through both quantitative and qualitative defects. Progressive depletion of CD4^+^ T cells—particularly naïve and central memory subsets—impairs coordination of adaptive immune responses. Functional exhaustion of CD8^+^ cytotoxic lymphocytes reduces clearance of virally infected or transformed cells. Concomitant B-cell hyperactivation and exhaustion compromise effective humoral immunity, and dendritic cell dysfunction—with impaired maturation and reduced MHC-I and co-stimulatory molecule expression—limits optimal priming of virus-specific T-cell responses [55]. Even under suppressive antiretroviral therapy, incomplete immune reconstitution and residual immune activation allow latent oncogenic viruses to escape full immunological control. In this setting, oncogenic viruses exploit weakened surveillance mechanisms. Insufficient cytotoxic control permits the survival and expansion of EBV-infected B cells, increasing the risk of lymphoproliferative disorders and non-Hodgkin lymphomas. Persistent immune dysregulation similarly facilitates HHV-8 replication, sustaining the angiogenic and inflammatory milieu characteristic of Kaposi’s sarcoma. Reduced clearance of high-risk HPV genotypes enhances viral persistence, integration events, and cumulative oncogene expression, promoting progression from intraepithelial neoplasia to invasive carcinoma. In individuals co-infected with HBV or HCV, sustained NF-κB activation and chronic pro-inflammatory signaling accelerate hepatic fibrogenesis and hepatocarcinogenesis, linking systemic immune dysfunction to tissue-specific malignant evolution [56,57,58]. Within this integrated framework, HIV may act as a viral oncogenic multiplier. By simultaneously enhancing viral persistence, creating a transcriptionally permissive inflammatory environment, promoting oxidative stress and replication-associated DNA damage, and impairing tumor immune surveillance, HIV amplifies the transforming capacity of co-infecting oncogenic viruses [59,60]. Malignant progression, therefore, reflects a multi-hit process in which viral oncogenes operate within an immune landscape persistently reshaped by HIV infection.

## 7. HIV Reservoirs and Oncogenic Microenvironments

A defining feature of HIV infection is the establishment of long-lived viral reservoirs that persist despite effective ART, predominantly within resting memory CD4^+^ T cells, tissue macrophages, and anatomical sanctuaries such as lymphoid aggregates and mucosal sites [61]. These reservoirs are not inert; they may contribute to a persistent state of immune activation and local inflammation, creating tissue microenvironments that inadvertently support oncogenic processes [62]. Within these niches, chronic antigenic stimulation drives sustained cytokine and chemokine production (e.g., IL-6, TNF-α, CCL2), which fosters recruitment and activation of myeloid cells and regulatory T cells, skewing the local immune milieu toward a pro-tumorigenic phenotype. Such environments resemble the inflammatory stroma observed in many virally mediated cancers, where elevated NF-κB and STAT3 signaling promote survival, proliferation, and angiogenesis [63]. Importantly, HIV reservoirs themselves can contribute to local immunosuppression by expressing viral proteins (such as Tat and Nef) that interfere with antigen presentation and effector cell function, further diminishing the capacity to eliminate transformed cells. In lymphoid tissues, persistent HIV expression drives follicular dendritic cell networks to retain immune complexes and apoptotic debris, potentially impairing germinal center dynamics and allowing oncogenic viruses like EBV to exploit niche dysfunction for aberrant B-cell expansion [64]. In mucosal tissues, resident macrophages and dendritic cells harbor both HIV and co-infecting oncogenic viruses (e.g., HPV), and local chronic inflammation disrupts epithelial integrity, facilitating viral persistence, epithelial–mesenchymal transition, and neoplastic progression [49]. Furthermore, metabolic reprogramming within HIV-affected microenvironments, driven by persistent inflammation and immune cell exhaustion, induces hypoxia-like signaling and alters lipid and glucose metabolism, conditions that have been implicated in supporting tumor initiation and progression across multiple tissues [54].

## 8. Immunosenescence and Immune Exhaustion in HIV Infection and Cancer

Immunosenescence refers to the gradual decline of immune function associated with aging, leading to increased susceptibility to infections, malignancies, and age-related diseases. In HIV infection, this process is accelerated by chronic antigen exposure and persistent viral replication, while in cancer it is driven by continuous immune engagement with tumor antigens. Chronic stimulation of immune cells, particularly via Toll-like receptors (TLRs) and T-cell receptor (TCR) signaling, triggers T-cell exhaustion and systemic inflammation, impairing the generation and maintenance of functional memory cells [65,66,67,68]. A hallmark of immune exhaustion in both HIV and cancer is the persistent expression of inhibitory receptors, or immune checkpoints, including PD-1, TIM-3, CTLA-4, and LAG-3 on T lymphocytes. Unlike transient expression seen in acute infections, this chronic upregulation progressively diminishes effector functions. Exhausted T cells lose proliferative capacity, reduce production of cytotoxic molecules such as granzymes and perforins, and eventually fail to secrete key cytokines, including TNF-α and IFN-γ, which are critical for tumor and virus-infected cell recognition and clearance. This hierarchical loss of function undermines immune surveillance and favors persistence of oncogenic viruses and early neoplastic lesions. TOX (Thymocyte selection-associated high mobility group box protein) is a master regulator of T-cell exhaustion. Chronic antigenic stimulation activates intracellular pathways that, through TOX, remodel chromatin accessibility, promoting sustained expression of inhibitory receptors while repressing genes responsible for effector functions and long-term memory. This epigenetic reprogramming stabilizes exhaustion, so that even after antigen clearance, T cells often fail to regain full functionality. In cancer, exhaustion frequently occurs in a heterogeneous “leopard spot” pattern within the tumor microenvironment, whereas in HIV it is more systemic, driven by viral persistence, CD4^+^ T-cell depletion, and chronic immune activation [66,67,68,69]. HIV-induced immune dysfunction extends beyond T cells. Regulatory T cells (Tregs) and myeloid-derived suppressor cells (MDSCs) expand during chronic infection, secreting inhibitory cytokines such as IL-10 and TGF-β. These cells suppress effector T-cell responses and contribute to a tumor-permissive microenvironment. Persistent immune activation also drives systemic inflammation, characterized by elevated TNF-α, IL-6, and IL-1β, which promotes angiogenesis, tissue remodeling, and proliferation of virus-infected or transformed cells. Chronic inflammation synergizes with immunosenescence and exhaustion to lower the threshold for malignant transformation. A clinically relevant example is Kaposi’s sarcoma (KS). Recurrent or refractory KS may occur despite effective ART and normalized CD4 counts. This is primarily due to persistent HHV-8 infection, incomplete restoration of virus-specific immunity, local immunosuppressive signaling, and residual HIV proteins such as Nef, which disrupt antigen presentation and enhance a pro-tumorigenic microenvironment. Immunological markers such as elevated IL-10 and altered cytokine profiles correlate with KS persistence and recurrence [69,70,71,72]. The combined effect of immunosenescence, immune exhaustion, expansion of Tregs/MDSCs, and chronic inflammation creates a permissive landscape for oncogenic viruses such as HHV-8, EBV, and HPV. In PLWH, this environment contributes to the elevated incidence of both ADCs and NADCs, despite effective viral suppression. Persistent HIV reservoirs in lymphoid tissues and mucosal sites further amplify this effect by maintaining local antigenic stimulation, chronic inflammation, and residual viral protein expression, which collectively support tumor initiation and progression [44,45,46,47,48,49,50,51,52,53,54,55,56,57,58,59,60,61,62,63,64,65,66,67,68].

## 9. Could HIV Cure Strategies Modify Long-Term Oncogenic Risk?

Emerging HIV cure strategies—such as latency-reversing agents (LRAs), broadly neutralizing antibodies, therapeutic vaccines, and other immune-modulatory approaches—aim to reduce or eliminate long-lived viral reservoirs in resting CD4^+^ T cells, tissue macrophages, and anatomical sanctuaries that persist despite suppressive ART [73]. Because these reservoirs contribute to chronic immune activation, local inflammation, and tissue microenvironments that facilitate viral oncogenesis, their reduction could theoretically decrease long-term cancer risk in PLWH [74]. For example, by diminishing the pool of HIV-infected cells expressing Tat or Nef, cure interventions may restore more effective antigen presentation, reduce NF-κB–driven pro-inflammatory signaling, and limit the persistence of co-infecting oncogenic viruses such as EBV, HPV, or HHV-8. However, some approaches, particularly LRAs that induce viral transcription, could transiently increase local inflammation or immune stress, potentially creating short-term pro-tumorigenic conditions. Current evidence remains largely preclinical, and longitudinal studies are required to determine whether HIV eradication or functional cure strategies translate into measurable reductions in malignancy incidence.

## 10. Conclusions

HIV-associated cancers result from a complex interplay of direct viral effects, immune dysfunction, oncogenic co-infections, and molecular alterations, including epigenetic and metabolic changes. Despite effective ART, PLWH remain at elevated risk for both ADCs and NADCs. A nuanced understanding of these mechanisms underscores the importance of targeted prevention and early detection strategies. Early initiation of ART to preserve immune function, routine screening for high-risk malignancies (such as cervical, anal, liver cancers, and lymphomas), vaccination against oncogenic viruses (HPV, HBV), and effective management of co-infections and modifiable risk factors are critical components of care. Furthermore, emerging insights into immune exhaustion, chronic inflammation, and viral reservoirs may inform future personalized interventions and immunotherapeutic approaches. By linking mechanistic understanding with practical strategies, this synthesis provides a foundation for optimizing long-term outcomes and reducing the cancer burden in PLWH.

## Figures and Tables

**Table 1:** Levels of Evidence for HIV Protein–Mediated Oncogenesis.

References	Clinically Validated Findings	Translational Data	Experimental Evidence (In Vitro/Animal)	HIV Protein
[19,20,21,22,23,24,25]	• Chronic inflammation and immune dysregulation linked to cancer risk; direct tumorigenic role not clinically confirmed	• Interacts with long noncoding RNAs in human BCBL1 cells (translational context)	• Modulates host proliferation/apoptosis via NF-κB, VEGF, bFGF in vitro • Affects choles-terol metabolism and KSHV replication in cell lines	**Tat**
[26,27,28,29]	• Persistent Nef expression correlated with immune dysfunction; no direct oncogenic causation shown in patients	• Evidence from human immune cell ex-plants suggesting impact on antigen presentation/translational immune sup-pression	• Alters signaling and immune pathways in vitro (Src kinases, PI3K/Akt) • Promotes oxidative stress and cytoskeletal changes in models	**Nef**
[30,31,32,33,34]	• Clinical evidence limited; Vpr contributes to persistent immune activation and reservoir effects linked to cancer suscep-tibility	• Vpr induced NF-κB activation linked to DNA damage signaling (translational)	• Induces DNA damage and G2/M arrest in vitro • Associated with genomic instability in preclinical work	**Vpr**
[35,36,37,38,39,40,41]	• Synergistic effect with co-infections and aging-related immune dysfunction, in-creasing cancer risk in PLWH	• Chronic immune activation; cytokine dysregulation; impaired dendritic and NK cell function; M2 macrophage polariza-tion	—	**Tat, Nef, Vpr**

Legend of Abbreviations: bFGF—basic fibroblast growth factor; BCBL1—body cavity-based lymphoma 1 cell line; G2/M—G2/M phase of the cell cycle; KSHV—Kaposi’s sarcoma-associated herpesvirus; NF-κB—nuclear factor kappa-light-chain-enhancer of activated B cells; PLWH—people living with HIV; VEGF—vascular endothelial growth factor.

## Data Availability

Not applicable.

## Abbreviations

The following abbreviations are used in this manuscript:

ADC/ADCs	AIDS-Defining Cancer(s)
ART	Antiretroviral Therapy
bFGF	Basic Fibroblast Growth Factor
EBV	Epstein–Barr Virus
HBV	Hepatitis B Virus
HCV	Hepatitis C Virus
HHV-8	Human Herpesvirus 8
HIV	Human Immunodeficiency Virus
HPV	Human Papillomavirus
KSHV	Kaposi’s Sarcoma–Associated Herpesvirus
KS	Kaposi’s Sarcoma
LANA	Latency-Associated Nuclear Antigen (KSHV)
LAG-3	Lymphocyte Activation Gene 3
LRAs	Latency-Reversing Agents
MDSC	Myeloid-Derived Suppressor Cell
MHC-I	Major Histocompatibility Complex Class I
NADC/NADCs	Non-AIDS-Defining Cancer(s)
NF-κB	Nuclear Factor Kappa-Light-Chain-Enhancer of Activated B Cells
NK	Natural Killer (cells)
PD-1	Programmed Cell Death Protein 1
PLWH	People Living With HIV
STAT3	Signal Transducer and Activator of Transcription 3
TCR	T-cell Receptor
TGF-β	Transforming Growth Factor Beta
Th	T Helper (cells)
TIL	Tumor-Infiltrating Lymphocyte
TME	Tumor Microenvironment
TIM-3	T-cell Immunoglobulin and Mucin-domain containing-3
TNF-α	Tumor Necrosis Factor Alpha
TOX	Thymocyte Selection-Associated High Mobility Group Box Protein
Vpr	Viral Protein R
Nef	Negative Regulatory Factor (HIV protein)
Tat	Trans-Activator of Transcription (HIV protein)
MSM	Men Who Have Sex with Men
